# Profiling immune cell-related gene features and immunoregulatory ceRNA in ischemic stroke

**DOI:** 10.1186/s43556-024-00237-4

**Published:** 2024-12-18

**Authors:** Yanbo Li, Sicheng Liu, Linda Wen, Linzhu Zhang, Xue Lei, Yaguang Zhang, Lei Qiu, Li He, Junhong Han

**Affiliations:** 1 Department of Gastrointestinal Surgery, Cancer Center and State Key Laboratory of Biotherapy, and Frontiers Science Center for Disease-Related Molecular Network, Laboratory of Gastrointestinal Tumor Epigenetics and Genomics, West China Hospital, Sichuan University, Chengdu, 610041 China; 2 Department of Neurology, West China Hospital, Sichuan University, Chengdu, 610041 China; 3 School of Basic Medical Sciences, Chengdu University of Traditional Chinese Medicine, Chengdu, 611137 China

**Keywords:** Ischemic stroke, Bioinformatics analysis, Immune cell, Immune-related genes, ceRNA

## Abstract

Molecules in immune cells plays a vital role in the pathogenesis of ischemic stroke (IS). The aim of this study is to profile the landscape of molecules on the basis of immune cells in IS peripheral blood and construct an immunoregulatory competing endogenous RNA (ceRNA) network. We collected and combined multiple public transcriptome datasets from the peripheral blood of IS patients and healthy controls. CIBERSORT deconvolution revealed that the proportions of CD8 and CD4 naive T cells, monocytes, and neutrophils changed significantly in the IS group. Intersecting the immune cell-related genes identified by weighted gene co-expression network analysis (WGCNA) and differential expression analysis, 38 overlapping candidate biomarkers were selected. Three machine learning algorithms, including least absolute shrinkage and selection operator (LASSO), support vector machine-recursive feature elimination (SVM-RFE), and random forest were applied, and 11 distinct immune cell-related genes were identified. We obtained the mRNA-miRNA and miRNA-lncRNA interactions from StarBase v3.0, and constructed a ceRNA network based on the differentially expressed mRNAs, miRNAs, and lncRNAs. The aberrant expression of HECW2-centered ceRNAs in the peripheral blood of in-house patients was validated using quantitative PCR. We also revealed that the expression of HECW2 was positively correlated with lncRNAs LINC02593 through miRNAs miR-130a-3p, miR-130b-3p and miR-148b-3p in cells. These results show that there are distinct immune features between IS patients and healthy controls. The ceRNA network may help elucidate the mechanism of immune cell-related genes in IS and may serve as a therapeutic target.

**Supplementary Information:**

The online version contains supplementary material available at 10.1186/s43556-024-00237-4.

## Introduction

Ischemic stroke (IS) is the fourth most burdensome neurological disorder [1] and the third leading cause of death worldwide [2], resulting from an abrupt blockage of the cerebral artery. To date, there are no acknowledged or authorized biomarkers for IS, and we are still making efforts to look for approaches for IS diagnosis and effective therapeutics. Advances in omics technologies have given us a new chance to better understand all the traits of IS. Transcriptomic data are remarkable resource for reflecting expression changes and have been widely applied to identify disease-associated signatures. At the transcriptomic level, the existing evidence has focused mainly on the comprehensive difference between incident IS and normal controls [3, 4], but little attention has been given to the precise function of the molecules in the immune system involved in IS to provide indicative markers for therapeutic targets.

The immune system plays critical roles in injury and recovery from stroke, on the basis of the conclusions of published omics studies or clinical studies on IS [5, 6]. To date, using omics technology, many molecular features have been described to predict patient risk of suffering from IS [7]. Nevertheless, omics in peripheral blood is not specific to the origin of the biomarker. During incident IS, ischemic cells trigger immune cell activation, inflammation, and programmed cell death. Immune cells are particularly important in regulating the immune and clotting systems in IS, ultimately resulting in core infarct growth, and expanding inflammatory injury to the entire ischemic territory. They can infiltrate the ischemic parenchyma from the peripheral circulation, contributing to the inflammatory environment, as early as the acute phase of IS [8]. Responsive diapedesis has been shown to affect central and peripheral tolerance in the context of IS, leading to altered outcomes [9]. It is crucial to identify new targets for IS by identifying signature biomarkers of immune cells. To identify specific and effective therapeutics for immune cell-related inflammation in IS, we focused on targeted biomarkers and the potential regulatory network on the basis of immune cells.

Emerging computational tools, such as CIBERSORT and xCell [10, 11], have been applied to quantify bulk gene expression profiles, and enable comprehensive and precise in silico measurements of immune cell infiltration based on real-world transcriptome data. Integrative analysis of RNA profiles revealed that differentially expressed diagnostic markers in IS were correlated with immune cell infiltration levels [12]. The competing endogenous RNAs (ceRNAs) network is a newly proposed regulatory mechanism involving coding RNAs, non-coding RNAs, and microRNAs (miRNAs). In particular, in IS, studies related to ceRNAs (including lncRNAs and miRNAs) revealed that these ceRNAs are enriched mainly in inflammation and immune-related signaling pathways among different brain cell lines and are of great clinical value in the immune mechanisms of IS detection and neuroprotection [13, 14].

Here, by employing advanced computational approaches, such as CIBERSORT, WGCNA, LASSO, SVM-RFE, random forest and others, we produced a more accurate molecular signature of hub immune genes specific to their cell type and their associated ceRNA network across large publicly available datasets in the IS population. A list of 11 core genes whose expression wes distinguishable between IS patients and healthy controls was identified. A ceRNA network was also established and the hub ceRNA pair were verified with in-house data. Experiments were also performed to validate our findings. Taken together, our findings provide fresh insights into the coordinated patterns of gene expression in immune cells with potential ceRNA regulators as targets, and these targets could be used to develop effective treatments for the pathogenesis of IS.

## Results

### Schematic workflow of this study

**Fig. 1:**
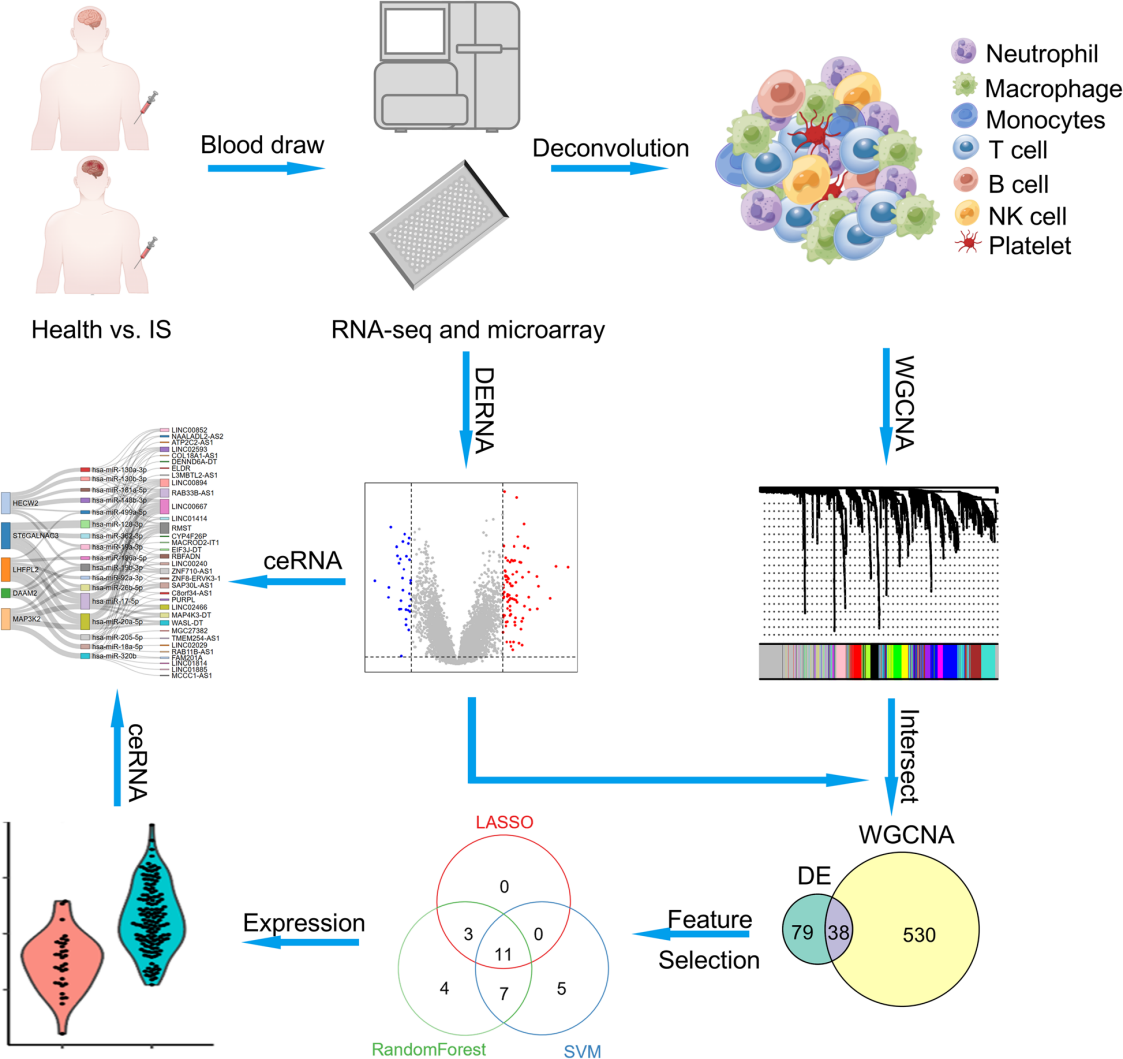
Schematic diagram of this study. RNA was quantified in the blood of ischemic stroke patients and healthy controls via RNA sequencing and microarray analysis. Differentially expressed RNAs, including mRNAs, miRNAs and lncRNAs were identified, and ceRNA networks were constructed. Differentially infiltrated immune cells were identified by deconvolution method. Most significant immune cells-related gene module were identified, and genes within these modules were intersected with DEmRNAs for LASSO regression, SVM-RFE, and random forest to filter core genes related to immune cells. Figure was created by Figdraw (www.figdraw.com)

The schematic workflow is shown in Fig. 1. Briefly, we downloaded publicly available datasets deposited at Gene Expression Omnibus (GEO), which included blood collected from IS patients or corresponding healthy controls, and performed either RNA sequencing or microarray analysis to quantify RNA expression. For mRNAs, there were 39 IS and 24 controls in GSE16561, 68 IS in GSE37587, and 69 IS and 23 controls in GSE58294. Overall, a total expression matrix of 176 IS and 47 controls were merged, followed by ComBat algorithm to ensure that the batch variants from these datasets were marginal (Fig. S1). For lncRNAs, the data of 5 IS patients and 5 healthy controls were extracted from GSE198710, and genes annotated to lncRNAs were retained for miRNAs, there were 20 IS and 20 controls in GSE110993, all of which were used for determining DEmiRNAs. We estimated the immune cell proportions via the deconvolution method from mRNA expression data and identified immune cells-related gene modules for differentially infiltrating cells. Genes within these modules were intersected with differentially expressed mRNAs (DEmRNAs), and LASSO regression, SVM-RFE, and random forest methods were subsequently performed to determine the signature that discriminates IS patients and controls. Using these DERNAs and the predicted mRNA-miRNA-lncRNA interaction sites, we constructed a ceRNA network with congruent expression. Finally, we performed experiments in the peripheral blood cells of IS patients from the stroke registry and in tool cells to validate the expression of immune-related hub genes and their regulatory ceRNA pairs.

### Differences in the immune profiles of peripheral blood between IS and controls

**Fig. 2:**
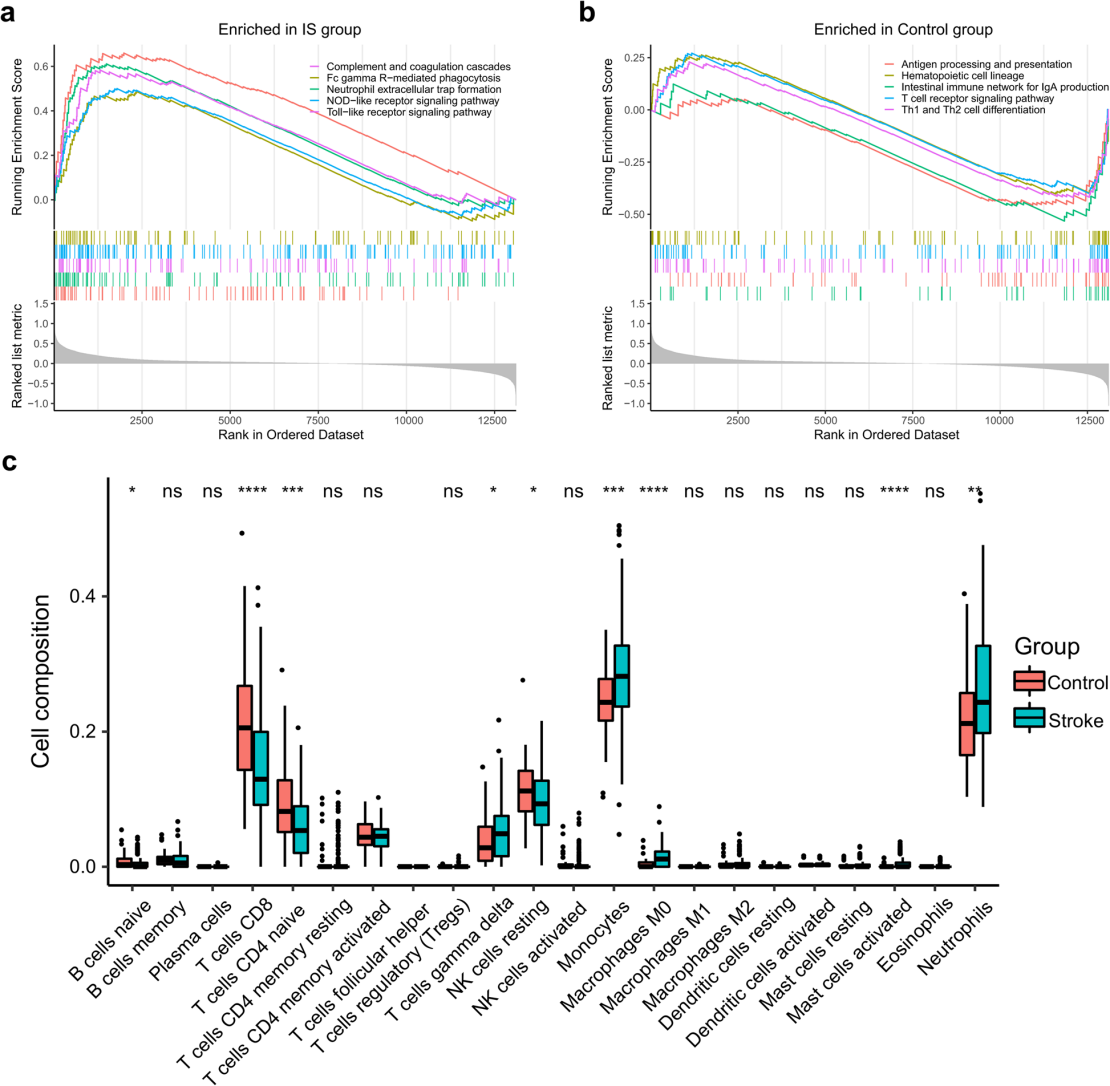
Dissecting distinct immune microenvironment in IS patients and healthy controls. **a** Gene sets from immune systems enriched in the IS group (*p* < 0.01, adjusted *p* < 0.05). **b** Gene sets from immune systems enriched in the control group (*p* < 0.01, adjusted *p* < 0.05). **c** The estimated composition of immune cells in IS patients and controls. In each box, the middle line represents the median value. The bottom and top of the boxes are the 25th and 75th percentiles (interquartile ranges), respectively. The scattered dots represent outliers. Significant differences between the two groups were calculated using Wilcoxon test. ns, not significant; *, *p* < 0.05; **, *p* < 0.01; ***, *p* < 0.001; ****, *p* < 0.0001

Given that the peripheral immune inflammatory response may play an important role in the pathogenesis of IS, we sought to determine distinguishable differences in the peripheral immunity between IS patients and controls. Gene set enrichment analysis (GSEA) was performed to determine different gene sets related to the immune system that were enriched in IS patients and controls (Supplementary Data 1). The top 5 gene sets of the IS were enriched in complement and coagulation cascades, Fc gamma R-mediated phagocytosis, neutrophils extracellular trap formation, NOD-like receptor, and Toll-like receptor signaling pathways (Fig. 2a), whereas the top 5 gene sets of the controls were enriched in antigen processing and presentation, hematopoietic cell lineage, intestinal immune network for IgA production, T cell receptor signaling pathway and Th1 and Th2 cell differentiation (Fig. 2b). To analyze the different compositions of immune cells in IS patients and controls, Wilcoxon test was used to compare the distributions of immune cells estimated with deconvolution method. We found that CD8 T cells and CD4 naive T cells were more abundant in the controls, while monocytes and neutrophils were more abundant in the IS patients (Fig. 2c). Clinical traits related to the immune landscape, including age and gender of the two groups, were displayed in Fig. S2. We also considered the time-dependent change in transcriptomic profiling by dividing the IS patients into two stages (samples collected within 0-24 h or within 24-48 h after IS). Both PCA results and compositions of the dominant cell types did not significantly differ between the two stages of IS (Fig. S3 a-b). Together, we revealed distinct immune profiles in the peripheral blood between IS and controls.

### Identification of immune cell-related gene modules by WGCNA

**Fig. 3:**
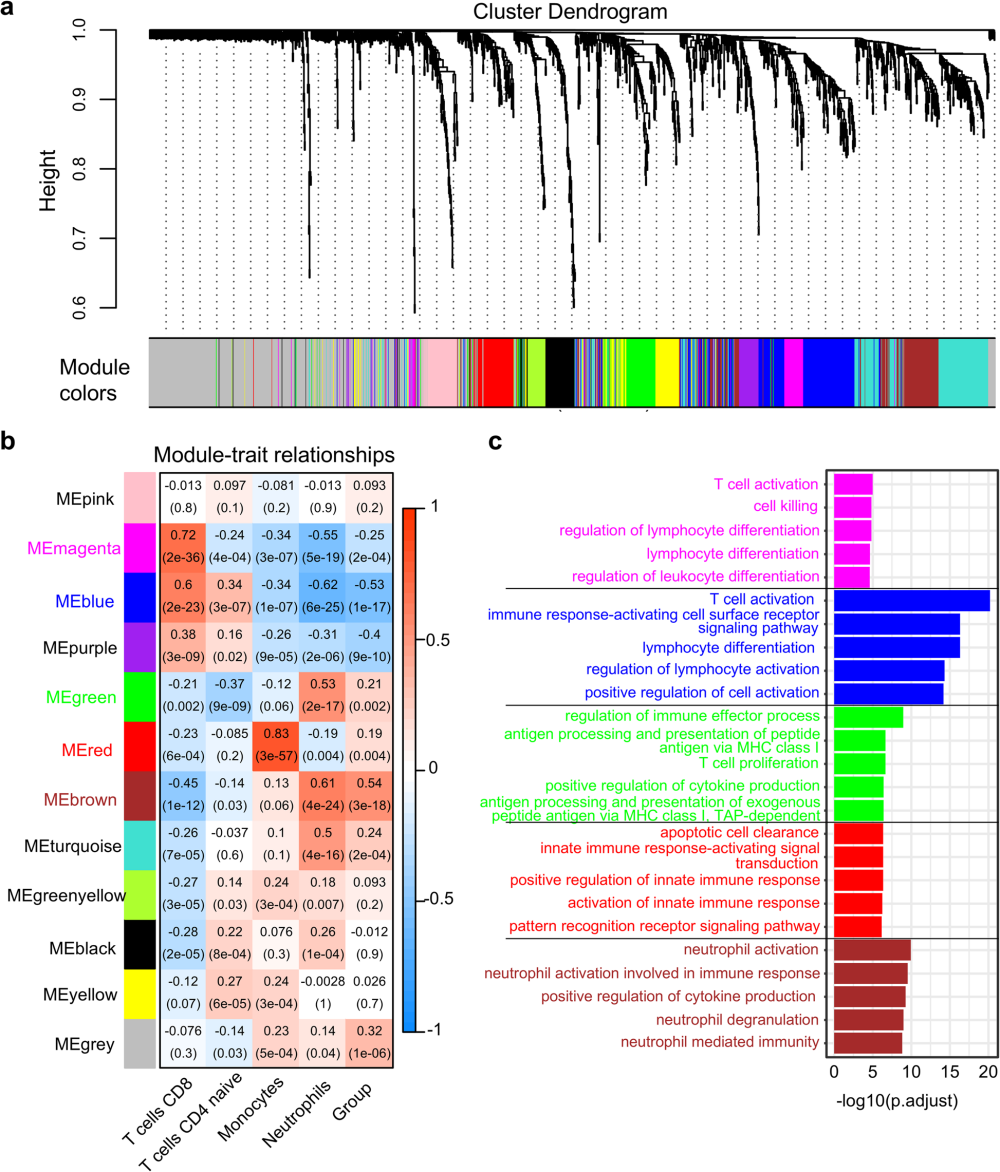
Selection of immune cell-related gene modules. **a** Clustering dendrogram of immune-related genes at the soft threshold of *β* = 5. Genes of the same color are assigned to the same module based on their expression correlation. **b** Gene modules related to significant immune cells as previously determined or groups (IS vs. controls). Each row represents a module, and each column represents a trait. The module color and upper number in each tabula indicate the correlation between the module and the trait, the lower number in brackets indicates *p* value. Significant immune cells-related modules were considered to have a correlation > 0.5 and *p* value < 0.05. **c** The top 5 enriched GO pathways associated with genes from each significant immune cell-related module. Terms were ranked by their -log10(p.adjust) values

To obtain genes associated with significant differentially infiltrating immune cells, WGCNA was carried out on a list of immune genes. Clustering dendrogram revealed that there were no obvious outliers (Fig. S4a). Several traits, including four immune cell types and disease status, were also matched to the sample dendrogram (Fig. S4a). Based on the scale-free network topology, the optimal soft threshold power was selected as 5 (Fig. S4b-c). Gene network was subsequently constructed to identify modules with the parameters minModuleSize = 30 and mergeCutHeight = 0.25. A total of 12 modules were recognized via the dynamic tree cutting method (Fig. 3a). According to the criteria Pearson correlation coefficient between the module and the trait > 0.5, *p* value < 0.05, genes in modules magenta and blue were closely correlated with CD8 T cells, genes in modules green and brown were linked to neutrophils, and genes in module red was highly associated with monocytes. Interestingly, genes in modules of blue and brown negatively or positively correlated with IS status, indicating that immune genes were differentially expressed at the onset of IS (Fig. 3b). There were 58 genes in magenta module, 205 genes in blue module, 87 genes in green module, 72 genes in red module and 146 genes in brown module, respectively (Supplementary Data 2). Notably, GO functional enrichment analysis revealed that genes within each module were strongly correlated with module trait. For example, genes in modules magenta and blue were enriched in T cell activations, and genes in module brown were involved in neutrophil activation, immune response, neutrophil degranulation, and neutrophil mediated immunity (Fig. 3c). These genes were defined as WGCNA genes for subsequent analysis. In summary, our findings indicated the good performance of immune cell-related gene module detection.

### Screening of immune feature genes in IS

**Fig. 4:**
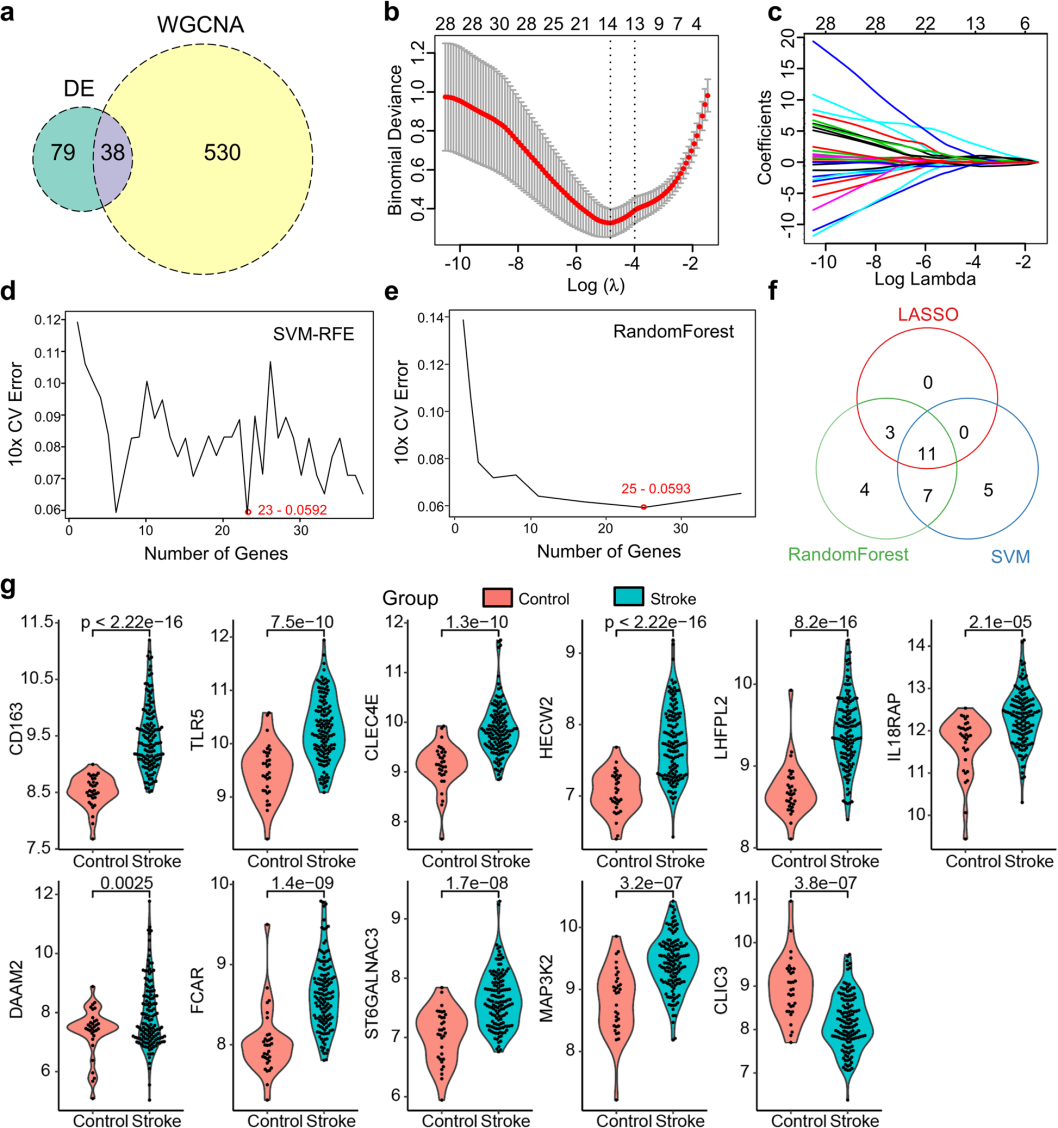
Selection of immune-related core genes. **a** Venn plot showing the overlapping genes between DEGs and WGCNA. **b** Plot of the cross-validated error rates. Red dot represents partial likelihood deviance, with error bars showing the standard error. **c** Plot showing the coefficients of overlapping genes regularized with log lambda. **d** Line plot showing the cross-validation error for various numbers of genes using SVM-RFE model. **e** Line plot showing the cross-validation error for various numbers of genes using random forest model. **f** Venn plot showing the overlapping core genes from LASSO, SVM-RFE, and random forest models. **g** Expression of selected immune-related core genes. Red: healthy control; blue: IS samples

We applied R package limma to identify DEmRNAs using the merged microarray data. A total of 117 DEmRNAs, including 85 upregulated and 32 downregulated mRNAs were detected between the IS patients and controls (Fig. S5a-b and Supplementary Data 3). Among the DEmRNAs and WGCNA genes, 38 overlapping genes were selected to identify core genes (Fig. 4a). All samples (*n* = 223) were randomly stratified into training set (*n* = 167, including 135 IS and 32 controls) and validation set (*n* = 56, including 41 IS and 15 controls). LASSO logistic regression algorithm yielded 14 immune-related genes robustly at the log(lambda.min) = -4.829 and log(lambda.1se) = -3.992 (Fig. 4b-c). While SVM-RFE and random forest methods yielded the lowest cross-validation error when the selected gene number was 23 and 25, respectively (Fig. 4d-e). As a result, 11 overlapping genes were detected by three algorithms as core genes, namely CD163, TLR5, CLEC4E, HECW2, LHFPL2, IL18RAP, DAAM2, FCAR, ST6GALNAC3, MAP3K2, and CLIC3 (Fig. 4f). We further investigated their expression and found that only CILC3 was significantly decreased in IS patients, whereas the other genes were upregulated (Fig. 4g and Fig. S6). In summary, the abovementioned genes were identified as the key genes differentially expressed in immune cells between IS patients and controls.

### Construction of ceRNA networks of IS

**Fig. 5:**
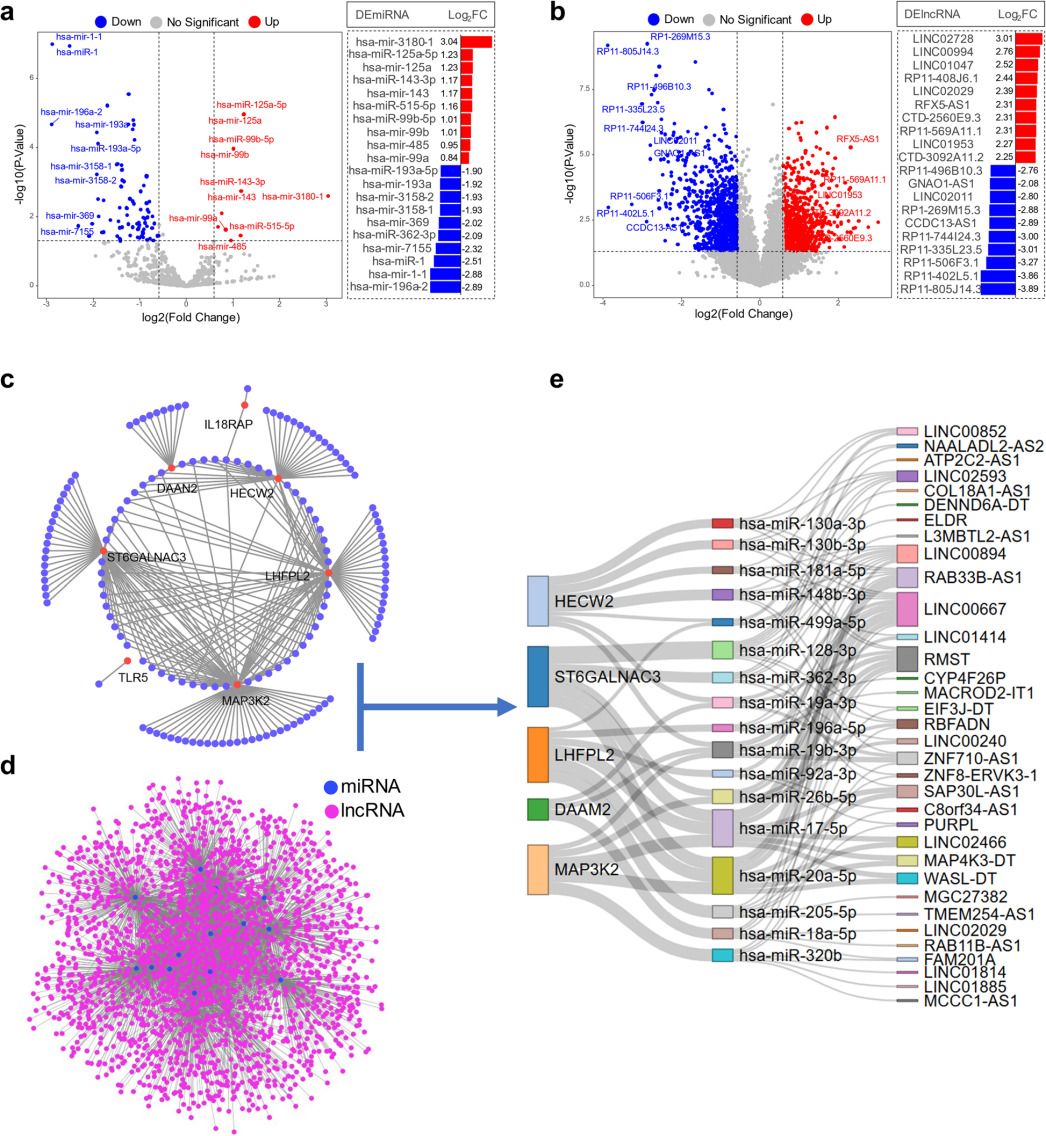
Construction of ceRNA networks for immune-related genes. **a-b** Volcano plots showing the differentially expressed miRNAs (A) and lncRNAs (B), respectively. Top10 up- and downregulated DERNAs are labeled along with log_2_(foldchange) in each panel. **c** The predicted mRNA-miRNA interactions using DEmRNAs. **d** The predicted miRNA-lncRNA interactions using DEmiRNAs. **e** Alluvial plot showing the concordant mRNA-miRNA-lncRNA expression pairs

We subsequently applied R package DESeq2 to screen out DEmiRNAs and DElncRNAs from the transcriptomic sequencing data. There were 13 upregulated and 115 downregulated miRNAs (Supplementary Data 4), 163 upregulated and 209 downregulated lncRNAs (Supplementary Data 5), a total of 128 and 372 DEmiRNAs and DElncRNAs were recognized (Fig. 5a**-**b). The DEmRNAs and DEmiRNAs were separately input into StarBase to predict potential mRNA-miRNA-lncRNA interactions (Fig. 5c**-**d). Finally, concordant mRNA-miRNA-lncRNA expression pairs were selected to construct the ceRNA network of IS (Fig. 5e). Our analyses revealed that a broad spectrum of ceRNAs were distinctly expressed during the onset of IS.

### Validation of hub ceRNA pairs by qPCR

**Fig. 6:**
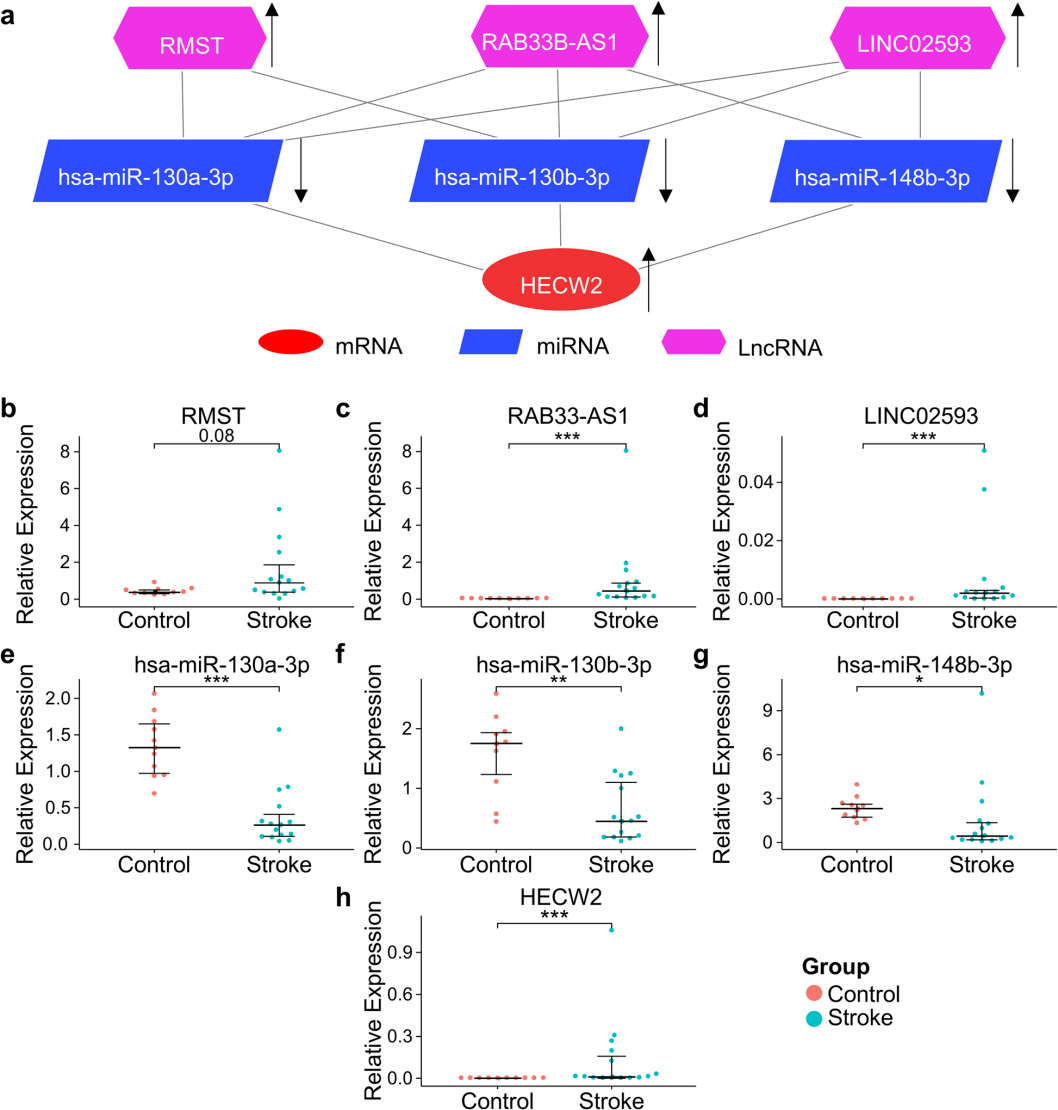
Validation of immune hub ceRNAs. **a** Hub ceRNA pairs that were detected by using the MCODE plugin in Cytoscape. **b-h** Verification of hub ceRNA pairs that was conducted by RT-qPCR analysis. Significant differences between the two groups were calculated using Wilcoxon test. *, *p* < 0.05; **, *p* < 0.01; ***, *p* < 0.001

The highly interconnected hub ceRNA pairs were identified via the Cytoscape plugin Molecular Complex Detection (MCODE), and included 3 lncRNAs (RMST, RAB33B-AS1, and LINC02593), three miRNAs (hsa-miR-130a-3p, hsa-miR-130b-3p, and hsa-miR-148b-3p), and one mRNA, HECW2. The expression of all lncRNAs and mRNA were upregulated in the peripheral blood of IS patients, while that of miRNAs were downregulated, which was consistent with the ceRNA hypothesis (Fig. 6a). The interaction sites between mRNA and miRNA, and between miRNA and lncRNA, suggested a potential regulatory mechanism in gene expression (Fig. S7). To further validate the aberrant expression of RNAs in IS patients, we collected blood from 15 IS patients within 72 h of onset and 10 age-matched healthy controls. The results demonstrated that the expression of 6 out of 7 RNAs was concordant, while the expression of RMST was not significantly increased in IS patients (Fig. 6b**-**h). Taken together, our experimental results generally confirmed that the ceRNA network worked in the acute stage of IS.

### Expression of Hecw2 in the peripheral blood of mice with middle cerebral artery occlusion (MCAO) at single cell resolution

**Fig. 7:**
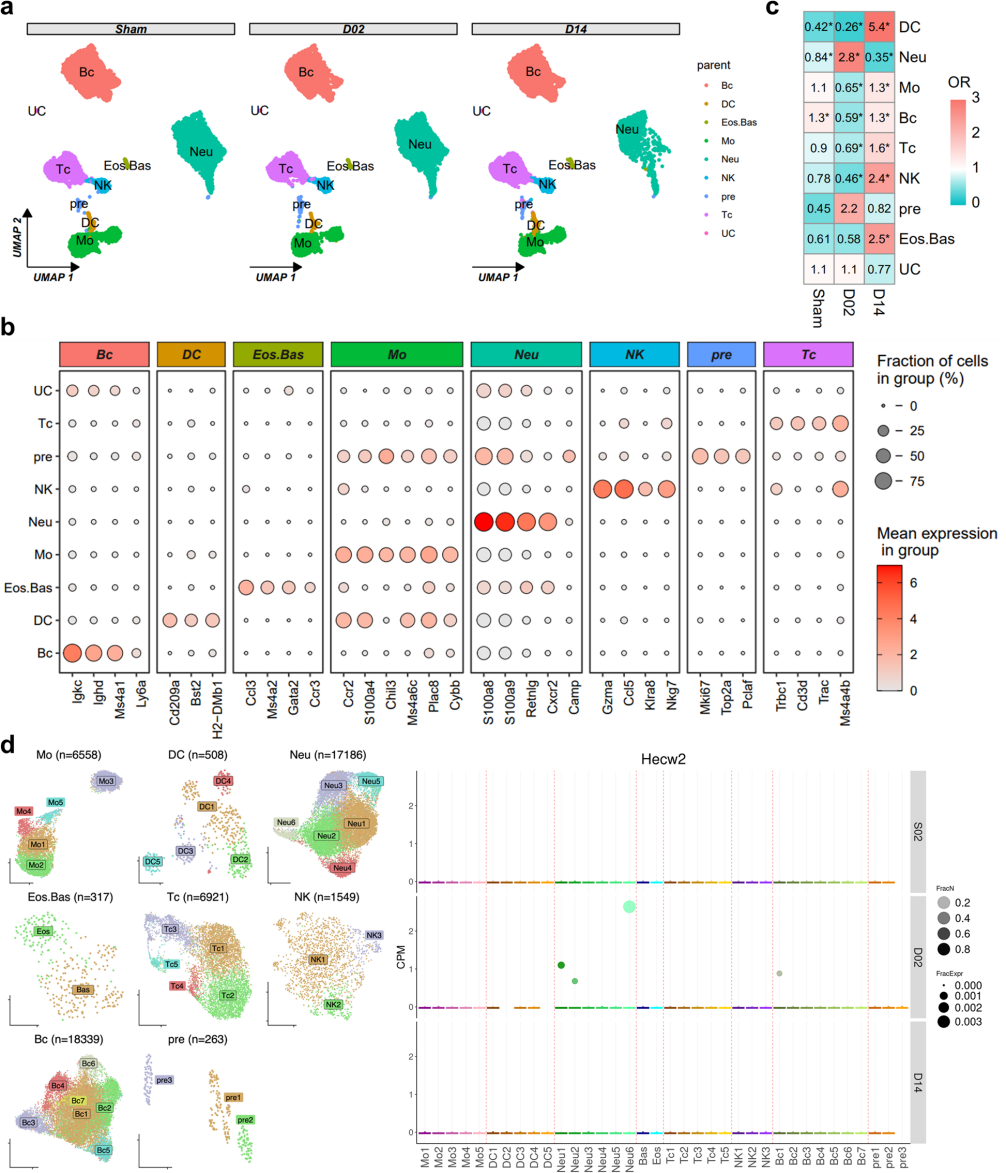
Exploring the expression of Hecw2 at single-cell level in mice models of experimental stroke. **a** Uniform Manifold Approximation and Projection (UMAP) showing the distribution of cells in each group. **b** Bubble plot showing the expression of marker genes for identification of each cell type. **c** Heatmap showing the OR of cells in each group. OR > 1.5 indicates that this cell is preferred to distribute in the corresponding group. *, *p* < 0.05. **d** Left, UMAP showing the number and distribution of subclusters of each cell type. Right, bubble plot showing the expression of Hecw2 in the identified cell subclusters in each group (accessed from https://anratherlab.shinyapps.io/strokevis/). Bc, B cells; DC, dendritic cells; Eos.Bas, Eosinophils-Basophils; Mo, Monocytes; Neu, Neutrophils; NK, natural killer cells; pre, hematopoietic precursors; Tc, T cells

With the advances in single-cell omics technologies, we wish to evaluate the expression of the core genes at single-cell resolution. A study by Cho YE et al. reported the circulating immune cell landscape in mild IS patients [15]. However, it is still not publicly accessible even after our request to the corresponding author via electronic mail. Thus, we used single cell RNA sequencing (scRNA-seq) data from mice after experimental stroke, namely, MCAO, in the acute and subacute phases from GSE225948 to support our previous results. According to the analysis procedures described in the original article [16], nine cell types were identified based on their marker genes (Fig. 7a-b). Consistently, we found that neutrophils accumulated in the peripheral blood of mice in the acute phase after stroke, while their proportion was decreased significantly in the subacute phase (Fig. 7c). After further subclustering these cells, we found that Hecw2 was predominantly expressed by neutrophil in the acute phase (Fig. 7d). However, owing to data limitations, we could not detect the expression of miRNAs or lncRNAs in the hub ceRNA pairs. These findings highlighted the importance of Hecw2 in neutrophils during stroke.

### The interaction between HECW2 and its ceRNA pairs

**Fig. 8:**
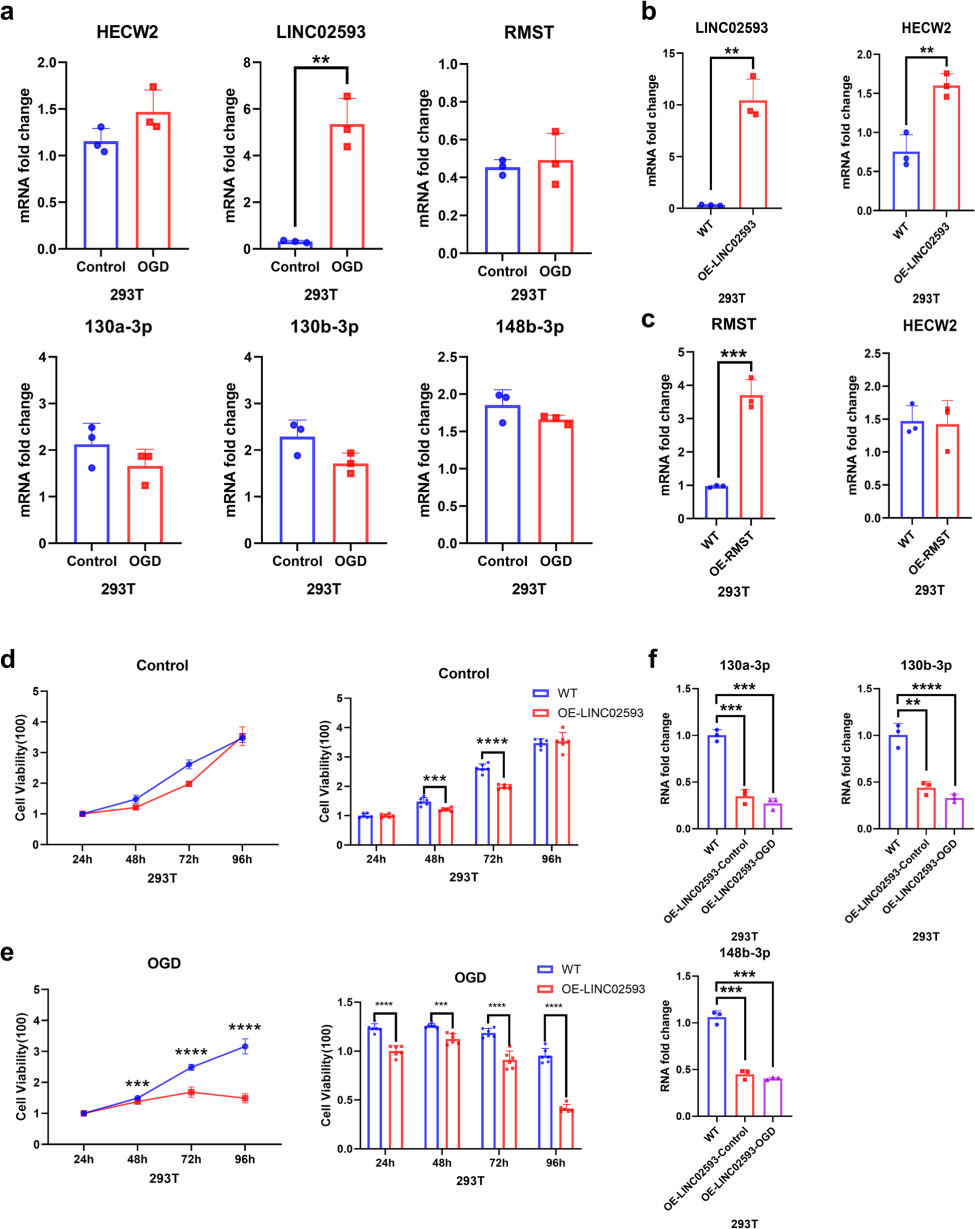
Relationships and mechanisms among HECW2, LINC02593 and RMST in HEK293T cells. **a** Fold changes in HECW2, LINC02593, RMST, hsa-miR-130a-3p, hsa-miR-130b-3p, and hsa-miR-148b-3p expression in OGD and normal controls. **b** HECW2 was upregulated compared with WT HECW2 because of the overexpression of LINC02593. **c** HECW2 did not change much after RMST was overexpressed. **d** Overexpressing LINC02593 had no effect on the viability of HEK293T cells in the normal control at 24 h and 96 h, but decreased it at 48 h and 72 h. **e** Overexpression of LINC02593 decreased cell viability in HEK293T cells subjected to OGD. **f** Compared with those in WT cells, the expression of hsa-miR-130a-3p, hsa-miR-130b-3p, and hsa-miR-148b-3p was downregulated by LINC02593 overexpression. *n* = 3 for all experiments, *, *p* < 0.05, **, *p* < 0.01, ***, *p* < 0.001, ****, *p* < 0.0001

To further verify the roles of HECW2 and its ceRNA pairs in IS, we used tool cells to illustrate the relationships among HECW2, LINC02593, RMST and microRNAs. Oxygen and glucose deprivation (OGD) is a classic model for ischemia in IS experiments. In the OGD model, the expression of LINC02593 was markedly elevated, whereas that of other genes, including HECW2, RMST, has-miR-130a-3p, hsa-miR-130b-3p, and hsa-miR-148b-3p, slightly changed but not significantly (Fig. 8a). When we overexpressed LINC02593 in HEK293T cells, HECW2 was upregulated compared with that in WT cells (Fig. 8b). However, HECW2 did not change much after RMST was overexpressed (Fig. 8c). We hypothesized that LINC02593 may play a dominant role in HECW2 modulation better than RMST does. Therefore, we focused on the function of LINC02593 in HECW2 regulation. LINC02953 overexpression had little effect on cell proliferation under normal conditions, but may decrease cell proliferation as the time extended to 48 to 72 h (Fig. 8d). When ischemia occurred, increased LINC02593 could wholly impair cell proliferation (Fig. 8e). The overexpression of LINC02593 also increased the RNA fold changes in the expression of hsa-miR-130a-3p, hsa-miR-130b-3p, and hsa-miR-148b-3p, in both normal and OGD models (Fig. 8f). These results suggested that LINC02593 had a regulatory effect on HECW2, possibly through the microRNAs hsa-miR-130a-3p, hsa-miR-130b-3p, and hsa-miR-148b-3p. But how LINC02593 works in neutrophils and the adverse effects of LINC02593 in neutrophils in IS remain to be further studied.

## Discussion

IS is one of the leading causes of long-term permanent disability with high incidence and mortality worldwide, severely damaging patients’ health and quality of life. Therefore, detecting and intervening in impending IS events or IS events in patients in the hyperacute stage is pivotal. Cerebral ischemia initiates a strong storm of gene expression in which immunoinflammatory responses are quickly activated contributing to the processes and progression of ischemic injury [17]. From the initial IS, various molecules, including brain-derived antigens, cytokines, and chemokines are secreted into the circulation system, and peripheral immune cells cross the blood–brain barrier to the injured site to stimulate immune responses [18, 19]. Several studies have reported that dynamic immune infiltration changes, immune-related gene dysregulation and pathway activation aberrations occur in the peripheral blood of IS patients [20, 21]. Hence, a comprehensive evaluation of different immune features for risk screening was performed. Emerging evidence has shown that early preclinical noninvasive diagnostic or therapeutic approaches based on molecular patterns will greatly benefit patients and provide opportunities for prevention [22–25]. However, these studies mainly focused on the whole-transcriptome data, which lacked cell- or molecule-type targeted analysis. The role of immune cell-specific genes had not been fully addressed. In addition, mRNAs, lncRNAs, and miRNAs played important antagonistic roles in IS occurrence and development in an antagonistic way. Although a systematic ceRNA network has been constructed via integrated analysis [26, 27], an immune cell-related gene-centered ceRNA network has not been proposed. Further validation of the creditability and reliability of the ceRNA network by using real-world data is also needed. In this study, for a reflection of IS, we extracted transcriptomic markers based on expression of immune cell-targeted genes in peripheral blood samples by performing integrated bioinformatic analysis on publicly available datasets. Based on hub genes, we constructed a ceRNA network and identified hub ceRNA pairs showing concordant expression. By validating the in-house data of patients and volunteers, we confirmed the aberrant expression of the HECW2-centered ceRNA pairs, which were distinguishable in the peripheral blood of IS patients and healthy controls.

In this study, we collected and reanalyzed a large cohort of IS patients and healthy controls. Pooling datasets allows for a larger, more comprehensive sample size, which can improve statistical power and help detect subtle biological signals that might be missed when analyzing smaller individual datasets. This approach enhances the robustness and reproducibility of the study, as findings supported by pooling datasets are less likely to be artifacts of small samples. However, pooling can also have potential limitations—particularly in the context of batch effects. Different platforms and technical variations across datasets can introduce biases that may confuse true biological signals. In this context, we applied appropriate normalization methods to minimize platform-specific biases for subsequent analysis. We first performed GSEA and CIBERSORT deconvolution method according to the transcriptome expression of IS patients and healthy controls to identify distinct activation of the immune pathway and circulating immune cells. Both methods revealed that neutrophils and their related pathway, such as neutrophil extracellular trap formation, were hyperactivated in IS patients, whereas various T cells and their relevant pathways, such as T cell receptor signaling pathway, and Th1 and Th2 cell differentiation pathways, were abundant and enriched in the healthy controls. As expected, neutrophils are the most abundant white blood cells in the circulation, and play a central role in innate immune defense [28]. The gene, HECW2, in neutrophils were significantly upregulated in the pathophysiological process in IS. These findings lay the foundation for further studies on interventions and prediction of IS. Researchers have indicated that neutrophil extracellular traps released by neutrophils impair vascular remodeling during stroke recovery [29]. Using flow cytometry, Wang et al. reported that the proportions of T-lymphocytes and natural killer cells in the circulation system of IS patients were remarkedly lower than those in healthy controls [30], which was consistent with our results. We also found that the proportion of monocytes increased after IS onset. These results are in line with known findings that proinflammatory signals from immune mediators promptly stimulate nearby cells and affect the infiltration of multifarious inflammatory cells, including neutrophils, monocytes/macrophages, different subtypes of T cells, and other inflammatory cells into the ischemic region to exacerbate brain damage [31]. All this evidence highlighted the effectiveness and reliability of in silico methods for identifying and interpreting biological conditions.

We next sought to determine the molecular patterns underlying the differences in immune cell infiltration between IS patients and healthy controls. WGCNA is a virtual approach that can calculate and summarize gene–gene correlations and use eigengene network methodology to uncover relationships between modules and external sample traits. In this study, WGCNA algorithm was applied to construct weighted gene network and explore the associations between divergent immune cell types and gene modules. As expected, genes within each module were functionally enriched in high module relationship-associated pathways. We further screened 117 DEmRNAs between IS patients and healthy controls, intersected them with 568 WGCNA genes, and ultimately identified 38 candidate biomarkers. LASSO is a supervised machine learning method used for feature selection and regularization of data models, and has yielded great prediction accuracy in many diseases, including cancer, COVID-19, and rheumatoid arthritis [32–34]. Based on LASSO logistic regression, SVM-RFE, and random forest models, eleven core genes was identified with good ability to distinguish IS patients from healthy controls. Among the 11 genes, Chloride Intracellular Channel 3 (CILC3) was the only gene with decreased expression in IS patients compared with healthy controls. CLIC3 is a protein-coding gene belonging to the chloride channel family that is located mainly at the cell membrane. It is correlated with the activation of cAMP-dependent PKA and hepatic ABC transporters. Some voltage-gated chloride channels have been shown to promote autophagy to protect ischemia reperfusion [35]. However, there is no evidence showing how CILC3 works in IS. Gene Ontology (GO) annotations related to this gene include chloride channel activity and voltage-gated chloride channel activity. Therefore, CILC3 may react to the injury caused by ischemia reperfusion. CD163, TLR5 and MAP3K2 have been studied for their role as potential biomarkers in IS patients. CD163 (OR, 2.283; 95% CI, 1.252–5.724; *p* = 0.03) was associated with the poor prognosis for IS patients [36]. TLR5 has been shown to influence the serum level of HDL-C in IS patients in Chinese Han population [37], and it may play a role in the control of hyperlipidemia as well as atherosclerosis. MAP3K2 is highly expressed in the serum of IS patients [38]. CLEC4E and IL18RAP have been shown to take part in the pathophysiological process of IS patients [39]. HECW2, LHFPL2, DAAM2, FCAR, ST6GALNAC3, and CLIC3 have not yet been reported. These genes are differently expressed in peripheral blood cells, and may take part in different processes in specific cell types. More studies are needed to determine who is closely related to IS in the pathophysiological process.

lncRNAs and miRNAs likely play important roles in the immune mechanisms of IS sponging to regulate immune-related gene expression. Hub genes of ceRNA networks had been demonstrated to be mainly enriched in inflammation and immune-related signaling pathways. Understanding the antagonistic role of the ceRNA network in IS is important to facilitate the identification of new treatment targets. In this study, we constructed a novel ceRNA network with immune cell-related genes and identified HECT, C2 And WW Domain Containing E3 Ubiquitin Protein Ligase 2 (HECW2)-centered ceRNA as the hub pair. Mutations in HECW2 were reported to be associated with neurodevelopmental delay and hypotonia [40], and intellectual disability and epilepsy [41], suggesting that it might play an essential role in the neural system. In our study, HECW2 had the same trend in IS patients as it changed in the public repository. The discrepancy in expression between our in-house and public IS cohorts may be due to the ethnicity, sex or other factors. Our experiments confirmed that LINC02593 played a dominant role in regulating HECW2. LINC02593 is a noncoding RNA that has been rarely studied. To date, few studies have shown that it correlated with meningioma, glioma and myofibroblast activation [42]. However, how it works or interacts with other molecules remains unclear. Several studies have shown that RMST promotes microglial cell activation, and that knockdown of RMST inhibits IS progression through suppressing neuronal apoptosis and oxidative stress [43, 44]. The RMST/HECW2 axis needed to be further studied in the incidence of IS in neutrophils. We also adopted qPCR validation for HECW2 and its ceRNA pairs in an in-house cohort of IS patients. Although primers have been tested several times for primer dimers, HECW2 was finally identified, which was obviously elevated in IS patients with increased expression of lncRNAs and decreased expression of miRNAs. Although we retrieved no direct experimental evidence supporting the relationship between IS and the other lncRNAs and miRNAs, according to the ceRNA concept and their sequencing and dysregulated expression in IS. There might be a real chance that they exert crucial functions by sponging other known IS regulators. The molecular function also needs to be validated at the protein level, addition to qPCR. In addition, validation of the other candidates within this network is highly important, and future experiments should be considered. Clinical detection of the expression of ceRNA pairs with in-house data would also open a new window into IS diagnosis, therapy and prognosis combining genetics and clinical traits, which better reflects global inflammation after IS on the basis of immune cells.

To investigate the connection of our results to those from single cell transcriptomics on IS, we additionally searched GEO database with keywords “single cell RNA sequencing and ischemic stroke” and found a publicly available scRNA-seq dataset GSE225948, which profiled the cell population of peripheral blood from mice model of experimental stroke. We found that neutrophils were preferentially enriched in the acute phase after stroke. Specifically, the expression of Hecw2 was increased in neutrophils in the acute phase compared with sham or subacute phase after stroke.

The present study had several limitations. First, not all the IS data at the transcriptomic level were available and integrated into our study because of the different measurements of gene expression. New approaches to comprehensively integrate heterogenous data could be further explored. Second, when collecting publicly available datasets, the unmatched age, as well as other demographic features, between IS patients and healthy controls may influence the expression pattern of immune cells, resulting in an overestimation of the difference between the two groups at the transcriptomic level and potentially compromising our interpretation. This problem should be carefully considered and solved when collecting our in-house samples in the future. Third, since there is no such scRNA-seq data from the peripheral blood of IS patients, investigations of the time-dependent changes in the expression of immune cells in the peripheral blood is limited. Fine monitoring of hospitalized patients is needed. We plan to address this issue soon. Fourth, the amount of our in-house data was relatively small, and verification with large cohorts would improve the rigor of the study. Fifth, the IS initiates a complex process in the peripheral circulation and central nervous system, and the ceRNA regulatory network is also comprehensive. In our analysis, we verified 7 hub genes in the ceRNA network that were representative of only parts of pathogenesis, and further in-depth studies are warranted for complementary trials on the remaining genes. Furthermore, as the mechanism by which ceRNAs in circulating immune cells undergo biochemical changes and genetic regulation under the triggering of IS is still unknown, more in vivo and in vitro experiments could be conducted for further tests. However, our findings provide new insight into targeted therapy for IS from the perspective of the immune cell regulatory network, and in the future, we will use more trials or experiments to strengthen the evidence of the multifaceted roles of immune cell-centered ceRNAs in IS.

In conclusion, we provided preliminary information on the molecular signatures of different immune cell types by integrative bioinformatic analyses. Through multiple machine learning methods, we identified 11 immune cell-related core genes that were aberrant expressed between the IS patients and healthy controls. These genes were further used for constructing ceRNA networks, among which HECW2-centered ceRNA network pairs presented the highest potential as biomarkers and promising treatment targets both and in the IS patient cohort.

## Materials and methods

### Dataset collection and preprocessing

The GEO database is a simple and convenient platform to archive and share various disease-related high-throughput data. In this study, we comprehensively collected mRNA, lncRNA and miRNA expression matrics from several datasets consisting of data from IS patients and healthy controls (accession numbers GSE16561, GSE37587, GSE58294, GSE198710, and GSE110993) [23, 45–48], detailed information was list in Table 1. For the microarray data, raw intensity values were preprocessed for background correction, quantile normalization and summarization by robust multiarray average method. Probes indicating nonunique genes were filtered out. The average expression values were taken if the same gene was captured by more than one probe. Batch effect was alleviated using ComBat function in sva package [49]. Only genes represented in multiple GPL platforms were retained. For RNA sequencing, genes with at least one mapped read were obtained for subsequent analysis. Clinical information of these patients, including age, gender and status was downloaded via R package GEOquery [50].

**Table 1:** Information of datasets enrolled in this study

GEO accession number	Platform	No. Cases	No. Control	Data type
GSE16561	GPL6883	39	24	mRNA
GSE37587	GPL6883	68	/	mRNA
GSE58294	GPL570	69	23	mRNA
GSE198710	GPL21827	5	5	lncRNA
GSE110993	GPL15456	20	20	miRNA

### Identification of differentially expressed RNAs

The empirical Bayes model implemented in package limma was used to identify DEmRNAs from microarray [51]. For RNA sequencing, the read count matrix was fed into DESeq2, followed by size factor and gene-wise dispersion estimation, fold changes shrinkage and differential expression testing [52]. The reference genome GRCh38 from GENCODE was used to annotate and select lncRNAs. All differentially expressed RNAs, including DEmRNAs, DElncRNAs and DEmiRNAs were determined to be significant based on the criteria |log_2_foldchange|> 0.585 and *p* value < 0.05.

### Functional enrichment analysis

GSEA was performed in R package clusterProfiler, with gene matrices ranked according to log_2_FC and Kyoto Encyclopedia of Genes and Genomes (KEGG) pathways as input [53]. Gene sets from the subclass “immune system” of category “organism systems” were selected to reveal distinct immune process alterations between IS patients and healthy controls. A *p* value < 0.01 and an adjusted *p* value < 0.05 were considered significant.

Three types of GO enrichment, namely biological process (BP), cellular component (CC) and molecular function (MF), as well as KEGG pathway of genes of interest, were enriched and annotated by clusterProfiler [53]. The top terms with *p* values < 0.05 were considered statistically significant.

### Estimation of immune cell proportions

CIBERSORT algorithm, which was based on a novel application of nu-support vector regression, was performed to estimate the relative proportions of 22 immune cells in the blood with gene expression data and parameter permutation test set as 1000 times [10]. Wilcoxon test was used to discriminate discrepant immune cell types between IS patients and healthy controls. To focus on the most important cells, we considered cell types with mean composition > 0.05 and estimated *p* value < 0.01 to be significantly responsible for the discrimination of IS patients from healthy controls.

### Weighted gene co-expression network analysis

We sought to identify immune-related genes that associated with significantly differentially infiltrating immune cells. A list of 2006 immune-related genes were kindly provided by Xing Huang (School of Medicine, Zhejiang University, Zhejiang, China). WGCNA was performed to identify hub immune modules [54]. First, a gene co-expression similarity matrix was produced by calculating the Pearson correlation coefficients between each pair of genes. The similarity matrix was subsequently transformed into an adjacency matrix to form a topological overlap matrix using the best pick-up soft threshold *β* = 5 (R^2^ = 0.867). Average hierarchical clustering was used to classify genes into several modules based on their connection strengths with a minimal module size of 30 and a merging cutoff threshold of 0.25. Finally, we determined modules whose correlation with traits was > 0.5 and whose *p* value was < 0.05 were immune cell related, and genes within these modules were defined as WGCNA genes.

### Feature selection by multiple machine learning algorithms

Genes associated with both DEmRNAs and WGCNA were intersected for feature selection. To choose the most distinguishable gene signature (hereafter termed core genes), LASSO regression, SVM-RFE, and random forest methods with ten-fold cross-validation were used. These processes were conducted with R package glmnet, randomForest and custom R scripts msvmRFE (https://github.com/johncolby/SVM-RFE), respectively.

### Analysis of scRNA-seq data from mice peripheral blood

To compare the expression of core genes at single cell resolution, scRNA-seq dataset was also searched in GEO. However, no publicly available scRNA-seq data from the whole blood of IS patients was found. Instead, scRNA-seq data from sham and MCAO mice were obtained from GSE225948 [55]. To indirectly reflect the reliability of the core genes described in our findings, we compared the gene expression data from the mice single cell transcriptome with those from our findings. Doublets were detected and removed by R package DoubletFinder [56]. The filtered data were batch effect alleviated by Harmony [57], and then processed by Seurat pipeline [58], followed by expression normalization, scale standardization, finding neighbors and finding clusters. To characterize the distribution preferences of cells, odds ratios (ORs) were calculated by 2 contingency table and Fisher’s exact test as previously described [59]. Results were visualized by build-in function from Seurat and ggplot2.

### Construction of competing endogenous RNA networks

According to the ceRNA hypothesis, lncRNAs indirectly regulate the expression of mRNAs by competitively sponging miRNAs. In this context, upregulated expression of lncRNAs leads to downregulated miRNA expression, resulting in upregulated mRNA expression, or vice versa. Potential regulatory networks for miRNA-mRNA, and miRNA-lncRNA interactions were downloaded from StarBase v3.0 (https://starbase.sysu.edu.cn/) by the Web API using custom script “curl ‘https://rna.sysu.edu.cn/encori/api/miRNATarget/?assembly=hg38&geneType=mRNA&miRNA=all&clipExpNum=1&degraExpNum=0&pancancerNum=0&programNum=1&program=PITA,miRanda&target=all&cellType=all’” and “curl ‘https://rna.sysu.edu.cn/encori/api/miRNATarget/?assembly=hg38&geneType=lncRNA&miRNA=all&clipExpNum=1&degraExpNum=0&pancancerNum=0&programNum=1&program=PITA,miRanda&target=all&cellType=all’”, respectively [60]. Based on DERNAs, the concordant lncRNA-miRNA-mRNA expression pairs were determined to construct the ceRNA networks, which were visualized with R package networkD3.

### Selection of the hub ceRNA network

Based on the maximal clique centrality algorithm, significant ceRNA pairs with strong interconnections within the whole network were identified and selected by MCODE, a plugin in Cytoscape. The parameter settings for MCODE were as follows: degree cut ≥ 2, K-core ≥ 2, node score cut ≥ 2, and maximum depth = 100.

### Patient enrollment

The study included 25 participants, 15 IS patients recruiting from a prospectively maintained database of consecutive first-ever IS patients in a high-volume tertiary stroke center (West China Hospital, Sichuan University) and 10 normal controls. IS was defined based on the World Health Organization (WHO) criteria as a sudden interruption in the blood supply of the brain with focal neurological deficits persisting longer than 24 h. The diagnoses were reconfirmed by brain computed tomography (CT) or magnetic resonance imaging (MRI). Blood samples were collected from all patients at admission, within 72 h after the onset of IS. The study was reviewed by the Ethics Committee of West China Hospital, Sichuan University (ethical review number: 2020(69)). Informed consent was provided by all participants.

### Quantitative real-time PCR

Total RNA from peripheral blood cells or 293 T cells were isolated with Ultrapure RNA Kit (CWBIO, China) using TRIzol reagent. The quality and quantity of RNA were measured by Nanodrop (Thermo Fisher, USA). The Mir-X miRNA First-Strand Synthesis Kit (TaKaRa Bio, USA) was then used for converting miRNAs and other RNA molecules into cDNA according to the manufacturer’s protocol. Quantitative PCR was conducted by using NovoStart SYBR qPCR SuperMix Plus (Novoprotein, China), running on a CFX96 Touch Real-Time PCR Detection Instrument (BioRad, USA). The primer was predicted through Primer3web version 4.1.0, and the number of cycles was set to 40. Primer pairs are listed in Table 2. miRNA values were normalized to U6 and other RNA values were normalized to 18s, via the 2^−△△Ct^ method.

**Table 2:** Primers for RT-qPCR

Primer pairs	Forward 5’ → 3’	Reverse 3’ → 5’
RMST	GTCTGCTTAGGGTGAAACAAA	GTCTCCGTGGTTCTATGGG
RAB33B-AS1	GGTTTCTTCAGCTTGCCGC	CCTTAGGCTGCAAAACTCGC
LINC02593	GTCGGGGTCTGCCCTAA	GCCCTCTGTCCTCTGGAA
hsa-miR-130a-3p	CAGTGCAATGTTAAAAGGGCAT	Provided by the Kit
hsa-miR-130b-3p	CAGTGCAATGATGAAAGGGCAT	Provided by the Kit
hsa-miR-148b-3p	TCAGTGCATCACAGAACTTTGT	Provided by the Kit
HECW2	GAAACCCCAGCCTTTTCTT	GTCAGGTACAGTGGCCGAT

### Cell culture

HEK293T cells were purchased from Shanghai Cell Bank of Chinese Academy of Sciences. The cells were cultured with 90% DMEM (HyClone, USA) high glucose medium, 10% fetal bovine serum, 100 U/mL penicillin and streptomycin in 37℃, 5% CO_2_ incubator. OGD model was established in a hypoxic incubator which was filled with mixed gas containing 94% N_2_, 1% O_2_, and 5% CO_2_ to induce OGD injury at 37 °C.

### Plasmids

The pCDH-LINC02593, pMD2G, and psPAX2 plasmids were used at a ratio of 2:1:3 to construct a virus containing LINC02593. HEK293T cells were transfected with the plasmids, and the lentiviral supernatants were collected at 24 h, 48 h and 72 h. Then, lentiviral mixture was used to infect HEK293T cells. After 12–16 h of incubation, the culture medium was replaced with fresh culture medium, and 2.5 µg/mL puromycin was added to the culture medium to select for positively infected cells. The pCMV plasmid for RMST was also constructed and used to transfect HEK293T cells.

### Cell proliferation 

Cell proliferation was evaluated by CCK-8 assay. Briefly, five time points were set: 0 h, 24 h, 48 h, 72 h, and 96 h. After seeding, cells were cultured continually for above time points. At each time point, CCK-8 reagent (TOPSCIENCE, China) was added. OD values were measured after incubation for 2 h at 450 nm.

### Statistical analysis

R software (version 4.0.2, https://www.R-project.org/) was used for statistical analysis. Results with two-sided *p* values < 0.05 were considered to be statistically significant unless otherwise specified above.

## Supplementary Information

Supplementary Material 1.

Supplementary Material 2.

## Data Availability

All data used in this study are obtained from public datasets and access are mentioned in the main text. Additional materials and codes are available from lead contact upon reasonable request.
