# Supplementary material for: Profiling immune cell-related gene features and immunoregulatory ceRNA in ischemic stroke
Source: Mol Biomed. 2024 Dec 18;5:72. doi: 10.1186/s43556-024-00237-4 (PMC11652561; doi:10.1186/s43556-024-00237-4)

Profiling immune cell-related gene features and immunoregulatory ceRNA in ischemic stroke

Yanbo Li[1,2#], Sicheng Liu[1#], Linda Wen[1#], Linzhu Zhang[3], Xue Lei[1], Yaguang Zhang[1], Lei Qiu[1], Li He[2*], Junhong Han[1*]

This file includes:
Supplementary Figures 1-7

Supplementary Figures

Fig. S1. Data pre-process using Combat function to remove batch effect. (A) PCA projection of samples before normalization. (B) PCA projection of samples after normalization.

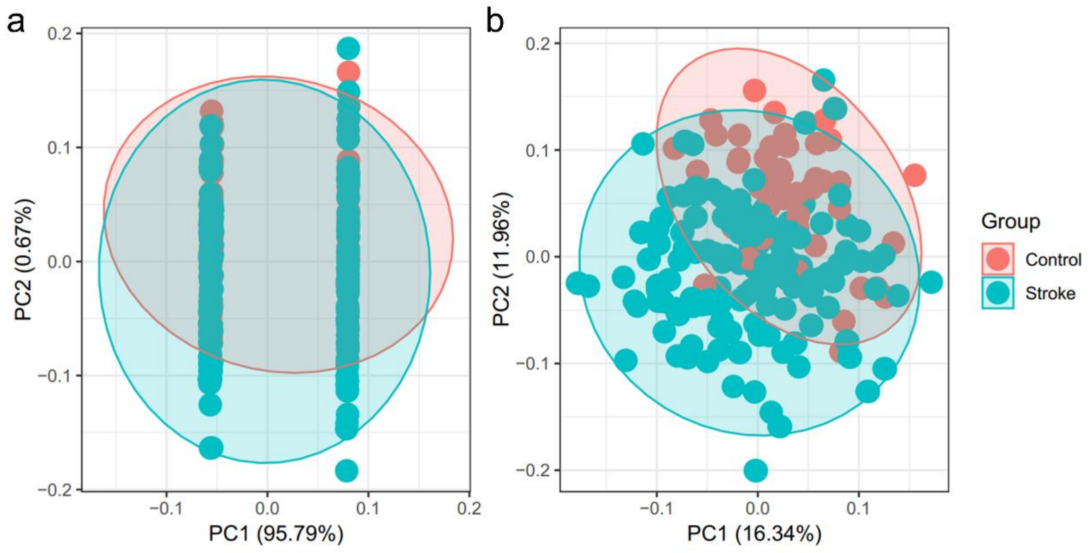

Fig. S2. The landscape of immune infiltration and clinical traits of IS and healthy control.

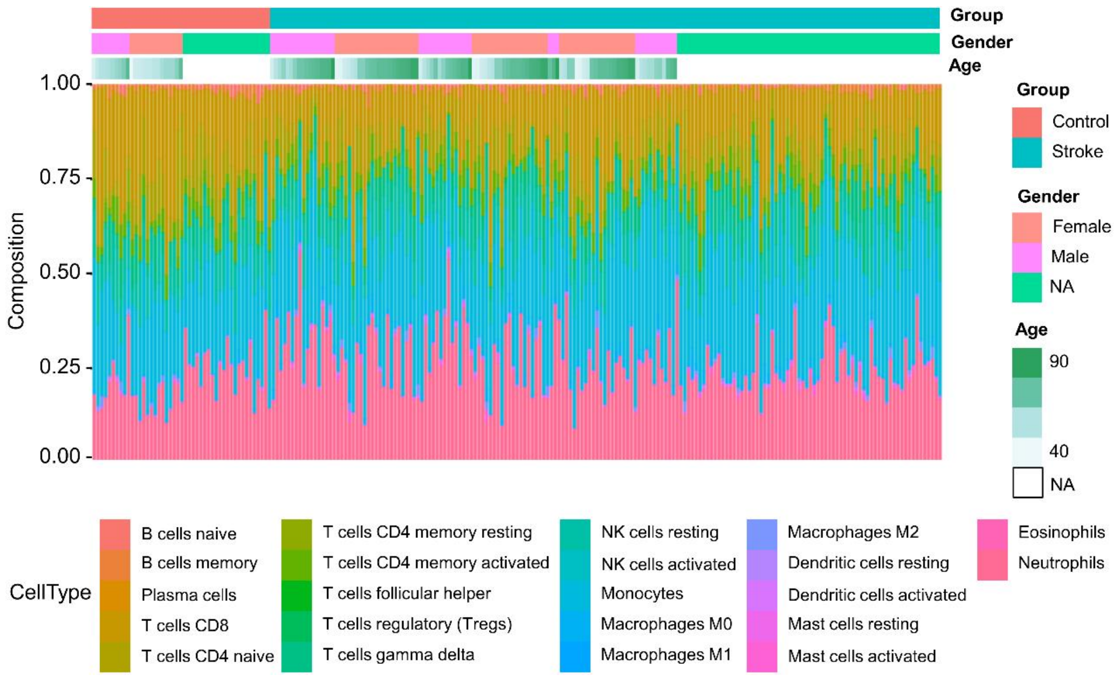

Fig. S3. Differences between samples collected within 0-24h and 24-48h after IS. (A) PCA projection of IS at different collection times. (B) The estimated composition of four immune cells in IS at different collection times and controls.

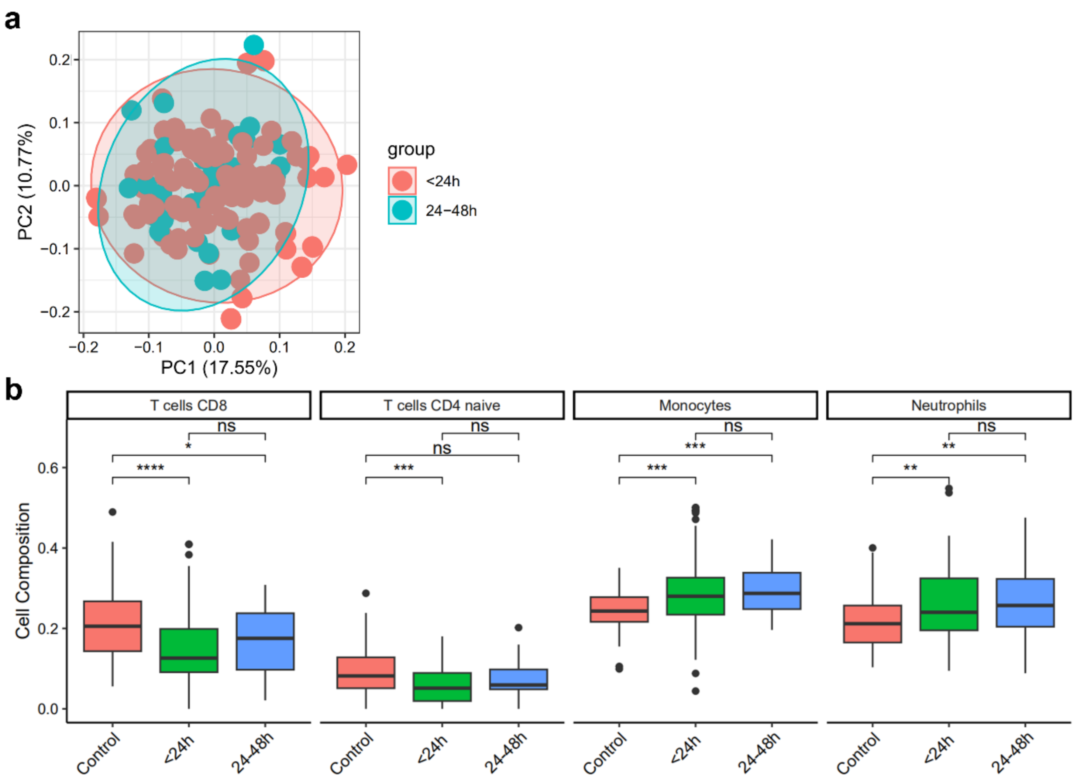

Fig. S4. Determination of optimal soft-thresholding power for WGCNA analysis. (A) Clustering dendrogram and trait heatmap of samples based on Euclidean distance. (B) The scale-free fit index corresponding to soft-thresholding power. (C) The mean connectivity corresponding to soft-thresholding power.

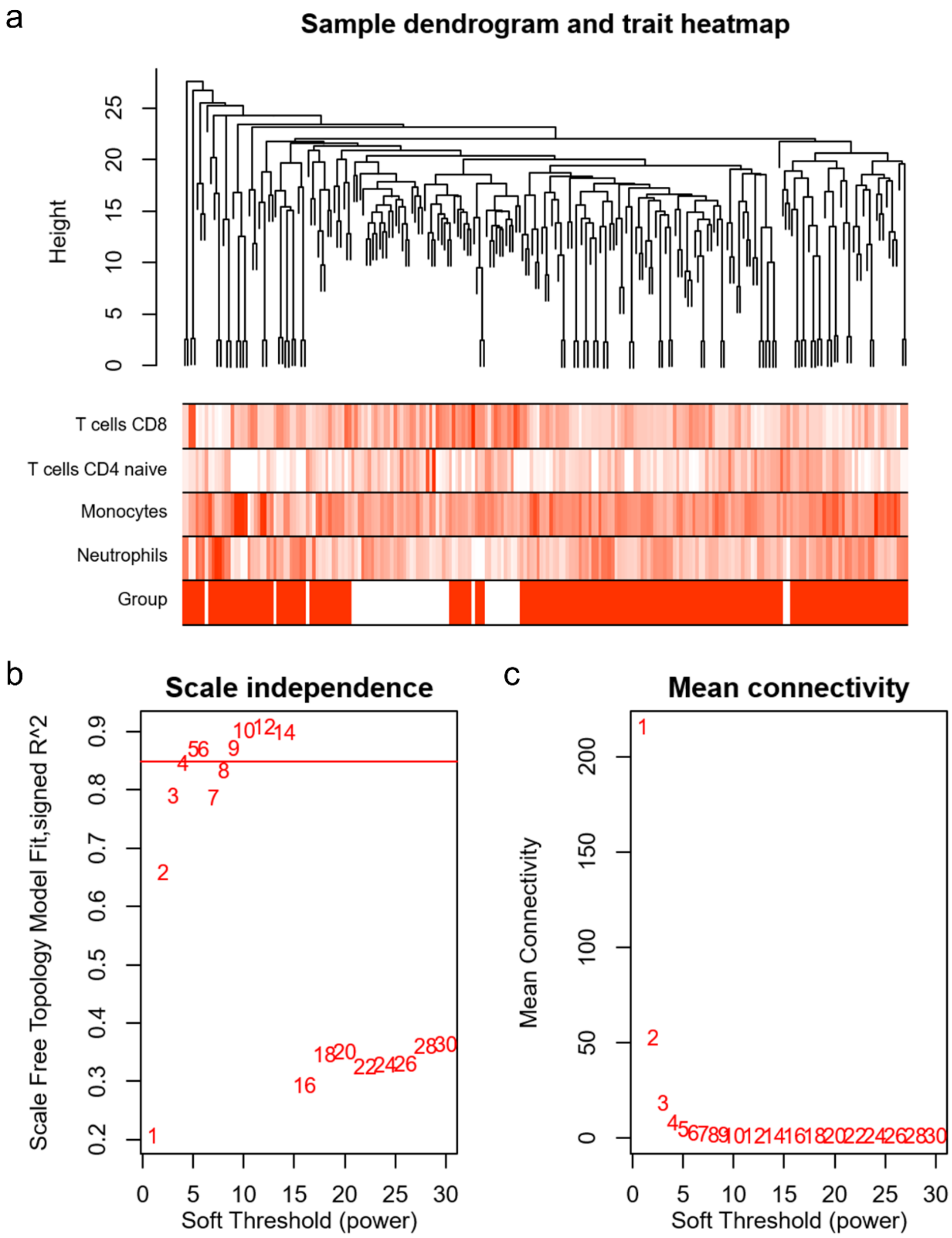

Fig. S5. Identification of DEmRNAs. (A) Volcano plots showing the DEmRNAs. Top10 up- and downregulated DEmRNAs were labeled along with log_2foldchange in the right panel. (B) Heatmap showing the expression of all DEmRNAs in each sample from IS and healthy controls.

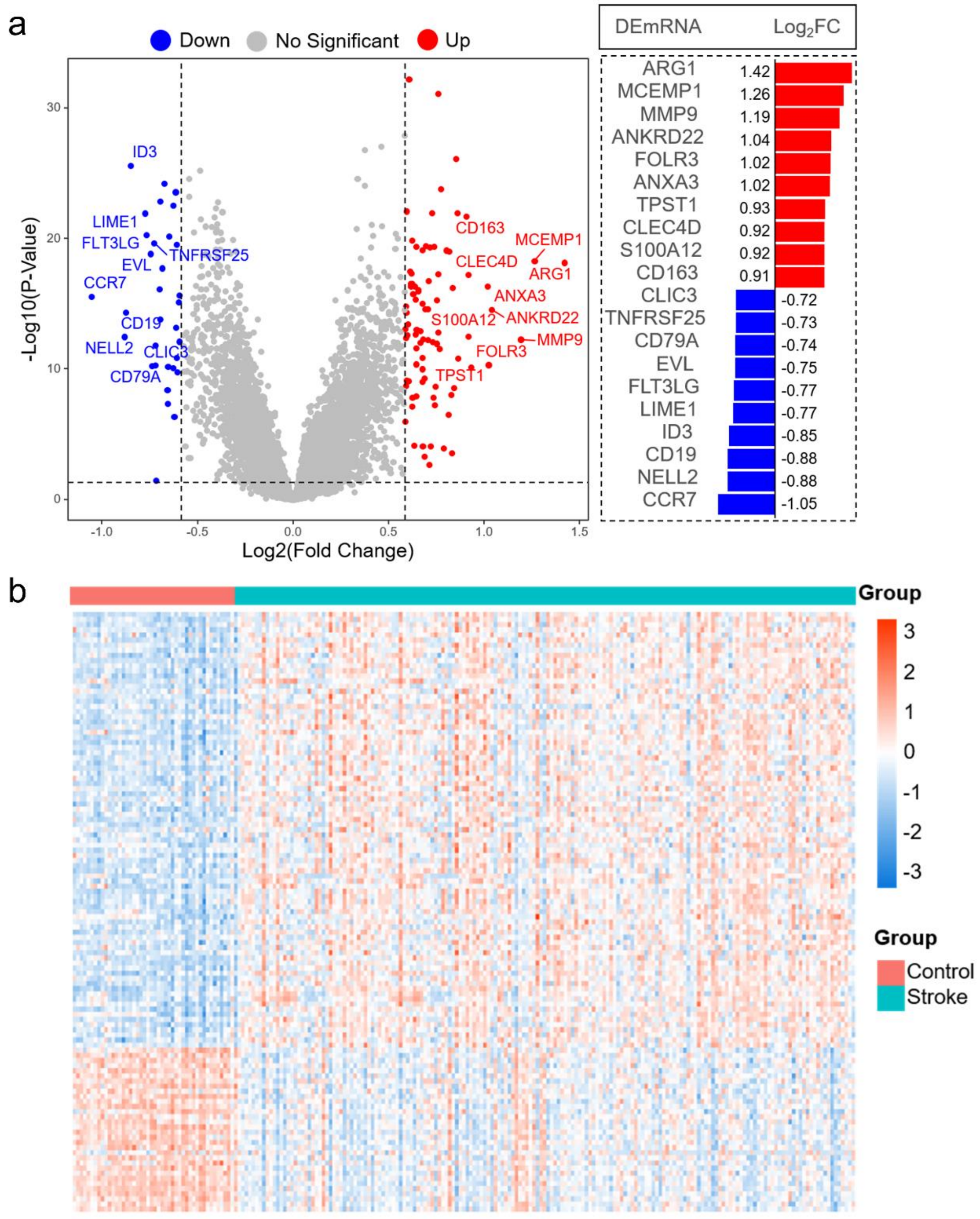

Fig. S6. Expression of selected immune-related core genes. Red: healthy control; blue: IS samples.

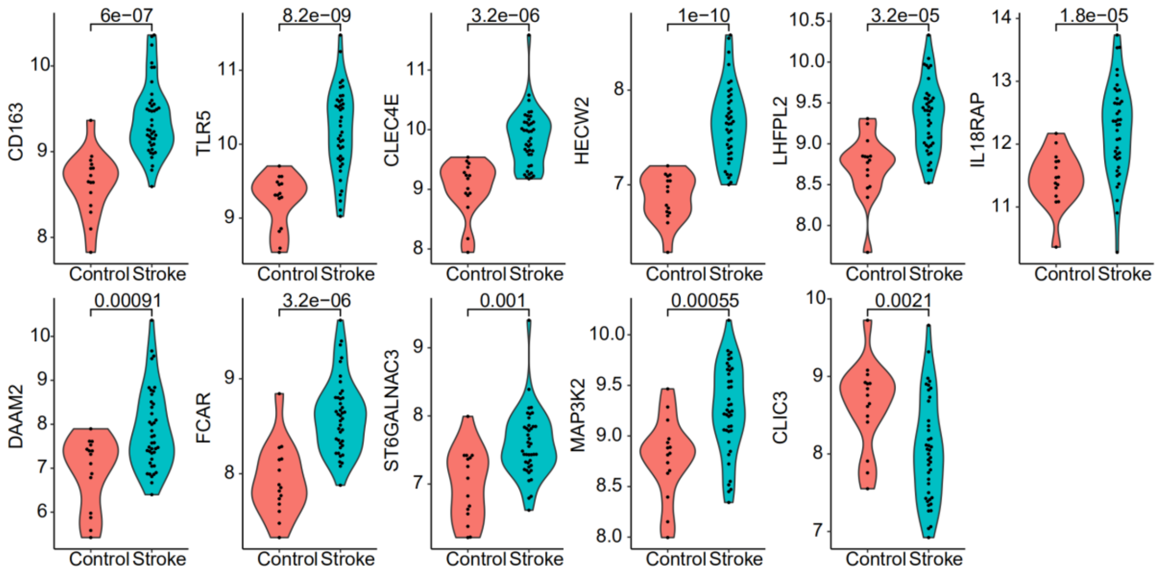

Fig. S7. The predicted interaction sites of hub ceRNA pairs.

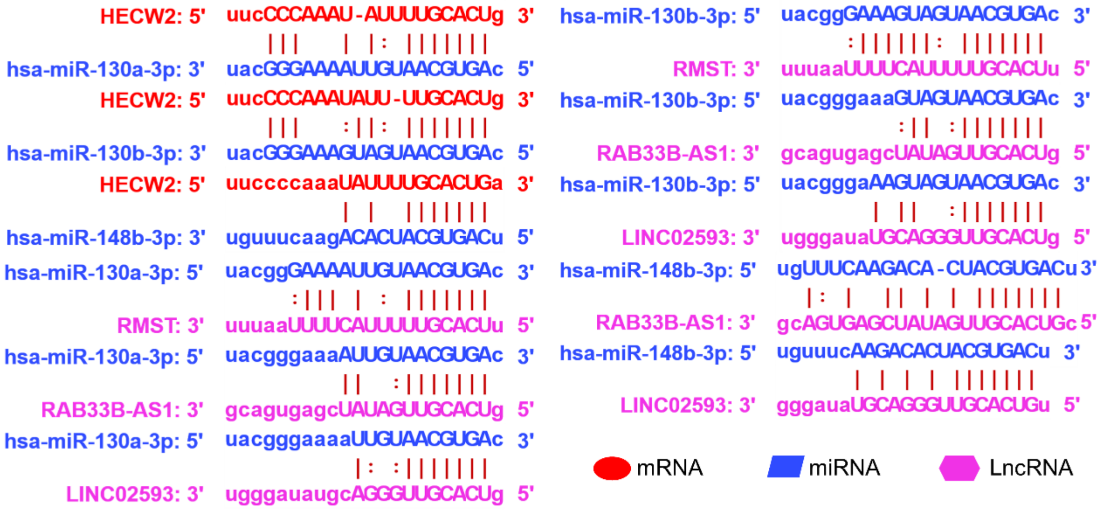

Supplement: Supplementary file 2 — Supplementary Material 2. [file 43556_2024_237_MOESM2_ESM.pdf]
